# The economic burden of chronic neurological disease

**DOI:** 10.1007/s00415-017-8632-7

**Published:** 2017-10-16

**Authors:** R. Wynford-Thomas, N. P. Robertson

**Affiliations:** Division of Psychological Medicine and Clinical Neuroscience, Department of Neurology, Cardiff University, University Hospital of Wales, Heath Park, Cardiff, CF14 4XN UK

## Introduction

The overall burden of neurological disease on society is considerable and complex with individual effects on social functions, employment and health care provision as well as secondary effects on family members and carers. Whilst a number of novel interventions offer great promise to patients, it is evident that there is a finite limit to resources available to health services for the introduction of new therapies. As a result, there is a growing focus on data that can contribute to the measurement of overall societal value of potential interventions.

Together with other metrics, economic data can be used to compare effects of new treatments on overall burden disease. These data can then contribute to statistical models that allow estimations of long-term treatment efficacy and quality-adjusted life years (QALYs). In countries that employ cost-utility analysis for health technology assessment, these models have a fundamental influence on decisions regarding drug availability and reimbursement. More recently, economic data have been particularly significant in multiple sclerosis (MS) as new disease-modifying therapies (DMTs) have become available. However, these data are equally relevant for other neurological diseases currently lacking effective treatment options, on which significant research and development resources are now focused.

The three papers discussed below explore some aspects of the economic burden of MS and Huntington’s disease (HD). In the first paper, the authors update economic data on the burden of MS as part of a Europe-wide study. The second paper looks at the cost of a clinical relapse in MS, whilst the third paper estimates the overall costs of HD.

## New insights into the burden and costs of multiple sclerosis in Europe: results for the United Kingdom

This study aimed to update estimated costs of all heath care and other resource utilisation related to MS. Data was collected retrospectively using standardised questionnaires as part of a cross-sectional retrospective study in 16 countries, collecting data on resource consumption, work capacity, health related quality of life and prevalent symptoms. Results were reported according to disability groupings measured by Expanded Disability Scale Status (EDSS 0.0–3.0 mild; EDSS 4.0–6.5 moderate; EDSS 7.0–9.0 severe).

Participants (*n* = 5928) were contacted by the national MS society and data analysed from a response rate that only reached 13%. Mean age of respondents was 56.7 years. Seventy-two per cent were below retirement age of which 36% were employed. Seventy-three per cent reported fatigue as the most bothersome symptom, with 22% reporting sick leave in the past 3 months, mainly related to relapse. Rates of employment fell rapidly with increasing disease severity, and of those unemployed patients, 57% reported MS as the main reason.

Unsurprisingly the total annual cost of disease per patient increased with disease severity. In mild disease health care costs dominated (overall mean cost: £11,400), whereas in more severe disease, production loss, informal care, investments and community services dominated (overall mean cost: £36,500). Informal care costs also rose from £585 to £11,337 in mild to severe disease. Mean cost of a relapse was estimated at £792 and all resource use increased during relapse, particularly inpatient and informal care. The authors concluded that the study data may be used to aid future design of health care provision and policy changes.

*Comment.* This broad ranging retrospective study investigated complex direct and indirect costs of health and exposes the complexities of understanding a number of interrelated factors. The respondent rate was very low despite two reminders, raising questions of how representative the data may be. In addition the study clearly has limitations in wider applicability since it focuses on a single health care service in the UK. However, these data usefully add to a wider perspective of a chronic and unpredictable neurological disease.

Thompson A et al. (2017) Mult Scler J 23(25) 204–216.

## Multiple sclerosis: relapses, resource use, and costs

Approximately 85% of patients with MS experience relapse as a characteristic clinical feature of their disease from onset. The effect of MS relapse and subsequent use of resources is highly variable and commonly depends on lesion site, duration of associated disability and other patient specific external factors. In this study, the authors aim to describe the range of health and social care resource use and costs according to the frequency, severity and duration of relapse to provide an estimate for cost of MS relapse.

Data were collected prospectively over 8 years from 1441 patients in the South West of England via standardised questionnaire with 11,800 questionnaires available for analysis. Patients already enrolled in an on-going longitudinal study addressing patient reported outcomes, were asked to retrospectively report details of up to four relapses over a 6-month period. Alternatively if the questionnaire was applied at the point of recruitment a 12-month period was considered. Data were collected for use of health and social care resources in the same period. Frequency, severity and duration of relapses were also recorded, along with treatment interventions and inpatient admissions.

Fifty-four per cent of patients had experienced a relapse during the reporting period, 30.9% reported no relapse and 14.9% were unsure. The main differences between resource costs in those that reported relapse versus those that did not, included use of a psychologist, GP, neurologist, MS specialist nurse or pain management service. Total mean cost in those that reported at least one relapse was £519, compared to £229 for patients who reported no relapse. Cost of relapse strongly correlated with relapse duration, with the greatest cost of relapse related to admission to hospital. However, an increasing number of relapses did not continuously increase resource use.

*Comment.* The authors have identified an important gap in the analysis of resource use in MS that has rarely been studied in detail. These data can be used to improve the understanding of cost information relating to relapses, but ultimately will have greatest value when studies such as this are standardised and applied over a number of countries and health care systems. Limitations once again revolve around the retrospective nature of the study and the self-reporting of relapse and disability, although the population seem representative.

Hawton AJ, Green C (2016) Eur J Health Econ 17:875–884.

## The societal cost of Huntington’s disease: are we underestimating the burden?

The worldwide prevalence of HD has been estimated at 5.7 per 100,000 with rates of 12 per 100,000 in northern Europe. Despite the considerable effect of the disease both on those affected as well as their families, there remains few data on the use of health and social care resources.

Data for UK patients were obtained from the European Huntington’s Disease Network (EHDN) REGISTRY, a longitudinal observational study which contains data on demographics, medical history and longitudinal data including a Client Service Receipt Inventory which contains information on hospital and residential services, primary and community care, diagnostic tests, informal care as well as aids and adaptations to the home. Patients were categorised by total functional capacity (TFC) score: stage I (the earliest stage) 11–13; stage II 7–10; stage III 3–6; stage IV 1–2; and stage V 0. Cost data were collected for service contact, medication, informal care, investigations and adaptations/aids. Data were available for 131 individuals, but did not include any stage V patients.

The largest increase in cost was observed in mean annual cost of informal care from stage III to IV (around £40,000). Mean annual cost of hospital/residential care also rose from £99.40 to £12,566 from stage I to stage IV. Mean annual costs per person in stage I were £2250 and rose to £89,760 in stage IV. Overall average annual cost per person was £21,605 equating to a national cost of £195 million per year.

*Comment.* This study identifies the high cost of informal care in HD which rises rapidly from stage III to IV. This may reflect the lack of effectiveness of outpatient-based care for these patients with higher levels of disability and the necessity to instigate more comprehensive community care. A detailed understanding of the overall economic burden of disease in HD will be crucial in future cost utility analysis of potential DMTs in HD. This study represents a good first step in developing data for these purposes as well as directing and planning health care for this vulnerable group of patients but will need to be considerably more detailed and include additional data on domains such as work and employment in order that the true costs of HD are not underestimated.

Jones C et al. (2016) Eur J Neurol 23:1588–1590.

